# Current level and determinants of inappropriate admissions to township hospitals under the new rural cooperative medical system in China: a cross-sectional study

**DOI:** 10.1186/s12913-014-0649-3

**Published:** 2014-12-18

**Authors:** Yan Zhang, Yingchun Chen, Xiang Zhang, Liang Zhang

**Affiliations:** School of Medicine and Health Management, Tongji Medical College, Huazhong University of Science and Technology, Wuhan, Hubei 430030 China

**Keywords:** Appropriateness evaluation protocol, Inappropriate admission, New Rural Cooperative Medical System, Rural China, Township hospital

## Abstract

**Background:**

The increased funding and reimbursement for the New Rural Cooperative Medical System (NRCMS) have provided residents in rural China with better access to inpatient services. This research aims to examine the level of inappropriate admissions to township hospitals under NRCMS, and the determinants that influence inappropriate admissions.

**Methods:**

A total of 2,044 medical records in 10 township hospitals were collected from five counties in Midwestern China by stratified cluster sampling and evaluated using the Appropriateness Evaluation Protocol (AEP), which was developed by a Delphi expert consultation of 32 experts. A two-level logistic regression model by MLwiN 2.30 was used to examine the determinants of inappropriate admissions.

**Results:**

Township hospitals had an average inappropriate admission rate of 26.5%. The highest rate of inappropriate admission was among patients aged more than 59 years old (30.1%). Inappropriate admissions mostly occurred for respiratory and circulatory diseases. Township hospital similarity and clustering were observed. Two-level logistic regression analysis showed that age, treating department, and disease were determinants of inappropriate admission.

**Conclusions:**

Township hospitals have a high rate of inappropriate admissions. Explicit diagnostic criteria and a standardized supervision system should be developed to reduce this.

## Background

Excess hospitalization demand means that patients receive unnecessary hospital services or those that are beyond their capacity to pay [1]. It can occur on three occasions: inappropriate admission, where patients receive unnecessary inpatient services rather than outpatient ones, or services that are beyond their capacity to pay; inappropriate inpatient services, in which patients receive inappropriate services during necessary hospitalization; and inappropriate level of care, in which patients are admitted to a higher-level hospital than necessary. This phenomenon increases the average medical expenditure per capita. This research focuses particularly on inappropriate admissions.

In 2003, the Chinese government launched the New Rural Cooperative Medical System (NRCMS), which had been implemented in all counties by 2005. The target population for the NRCMS was rural residents, who account for more than 60% of the population of China [2]. Rural residents voluntarily enrolled as families. The scheme’s sources of revenue included government subsidies and individual premiums. The population covered increased from 80.79 million in 2004 to 831.63 million in 2011, or 95.3% of the rural population of China [3].

Rural residents’ access to healthcare rapidly improved under the NRCMS. From 2003 to 2011, rural hospital admissions increased by 2.5 times from 3.4% to 8.4% (compared with the 0.3% increase from 1993 to 2003), the outpatient rate increased from 13.9% to 15.3%, and hospital delivery rates averaged from 68.9% to 95.9%. Simultaneously, the rate of unmet outpatient care declined from 48.9% to 5.4%, and the rate of unmet inpatient care declined from 29.6% to 17.3% [4]. At the same time, the caesarean section rate sharply increased from 19.2% to 36.3% [5]. Some studies have reported that inappropriate admissions have significantly contributed to this increase [6,7]. Many hospitals may admit patients who could have been treated as outpatients to increase the reimbursement from NRCMS, and patients often prefer to receive services as inpatients [7].

The consumer healthcare utilization model provides four steps for an inpatient procedure: assessing whether the patient needs healthcare; selecting a hospital; receiving the doctor’s proposal for diagnosis and treatment; and providing the care [8]. Although patients have absolute choice in the treatment process, they always take account of doctors’ proposals. Inappropriate admission therefore results from the joint decision of patients and healthcare providers [9]. In China, doctors have two possible reasons for causing inappropriate admission. The first is to avoid potential medical risks of outpatient care, because hospitalization allows for more examination and treatment to control the disease [10]. The second is to generate more personal revenue, because most doctors generate revenue based on the volume of medical care they have supplied [11]. Patients prefer hospitalization to outpatient care for three reasons: (1) cost saving—because hospital reimbursement comes from a pooled social fund, patients only pay a minimal amount personally; (2) to receive a more comprehensive and safe diagnosis and treatment than they would as outpatients [12]; and (3) convenience—repeated outpatient attendances, particularly for older people who live alone, are inconvenient in rural areas [13]. At present, both patients and doctors can benefit from inappropriate admission, so a preference by either side will usually lead to inappropriate admission for example, a doctor may choose to satisfy a patient’s inappropriate request [10,14].

There are many studies about the determinants of inappropriate admissions. Mozes et al. [15] found that inappropriate admissions were much higher in groups requiring an operation and in children than other groups. De Marco et al. [16] also demonstrated that inappropriate admissions were seen more often in children, especially if they had an influenza-like illness. Barker Bausell [17] found that older people also had a high inappropriate admissions rate. Pileggi et al. [18] demonstrated that emergency patients were at high risk of inappropriate admissions. Brown [19] found that the insurance reimbursement ratio and day limits on insurance payouts also had a significant influence on the admission appropriateness.

Two methods are internationally accepted in evaluating admission rationality, explicit diagnosis-specific criteria (EDC) [20] and implicit criteria [21,22]. The classification of disease diagnosis is not included in EDC. Instead, this provides reviewers with common criteria for all types of diagnosis, including the severity of the disease, the strength of services, and the patient’s need for hospitalization [23]. The alternative, implicit criteria, requires senior clinical doctors to review medical records and determine whether hospitalization was appropriate. Researchers need not supply any information; the reviewer’s judgment will depend entirely on their knowledge, experience, and skills [24]. EDC is often used by managers and other specialists in non-clinical medicine, and implicit criteria are employed by clinical medical experts. The results of the latter method are more reliable. However, insurance companies have limited funding and time, authoritative clinical medical experts are lacking, and doctors’ judgments on records vary, even for the same doctor at different times [23]. Using EDC is therefore more practical.

In many countries, an Appropriateness Evaluation Protocol (AEP), a form of EDC, is commonly used to assess the appropriateness of admission and hospitalization. By reviewing the patient's medical records according to explicit criteria, the AEP enables evaluation of the rationality of the care. This is based on medical and technical standards and is independent of disease category. An AEP review focuses on specific services or patients, instead of a specific diagnosis, so evaluation can be performed by non-doctors, and even used for undefined or incorrect diagnosis. This method is commonly used by insurance agencies for supervising hospitalization services.

In 1967, the United States implemented AEP admission and hospital standards [25]. In 1993, Austria, France, Italy, Portugal, Spain, Sweden, and the UK established a unified AEP standard for EU countries [26,27]. In 1994, a panel of subsidiary hospitals of Maastricht University in the Netherlands launched the US-AEP for medical insurance payments for hospital services [28].

Inappropriate admissions considerably increase hospitalization expenses [29] and account for 20% to 40% of operation days [30]. Ignoring inappropriate admission would hinder government inputs in public health as well as individual well-being [29].

The existing studies on excess demand for hospital services are more concerned with inappropriate services than hospitalization in China. NRCMS management agencies have formulated several policies to address inappropriate admissions, such as the creation of clinical pathways [31]. Nevertheless, standardized clinical pathways cannot be implemented easily in township hospitals because of the considerable variation in the service capacities of rural institutions. Some NRCMS management agencies consider an admission inappropriate if the duration of stay is less than 3 days, or the total cost of non-surgical hospitalization is less than 200 RMB. However, such rigid judgment criteria are likely to cause mistakes. Other NRCMS management agencies have formed panels to assess such matter, which means higher costs. There is no tool like AEP in China.

We aim to make clear the level and determinants of inappropriate admissions in Chinese township hospitals by establishing AEP criteria. This study will also help policy makers and health planners predict and plan for future needs.

## Methods

### AEP criteria

We used a Delphi expert consultation to build AEP criteria for township hospitals in rural China. All current judgments on clinical indicators were screened based on current AEP criteria in the US [25], the seven aforementioned EU countries [27], the Netherlands, and other countries [32], and China's current clinical pathway and literature studies. We then consolidated and classified the systems to develop a preliminary evaluation of AEP entries. A total of 32 experts were chosen to conduct three rounds of consultation and adjustment. Of the 32, 11 were from top-class national hospitals, 15 from county hospitals, four from township hospitals, and two from NRCMS management agencies. The experts’ deep understanding of China’s existing services in rural areas enabled them to assess the suitability and other indicators of systemic AEP standards, and so build the AEP criteria for inappropriate admissions to township hospitals.

The experts reviewed 400 randomly selected medical records from a township hospital to formulate two standards, a final AEP standard and implicit diagnostic criteria for clinical experts. Both were tested with a kappa test.

The study protocol conformed to the ethical guidelines of the 1975 Declaration of Helsinki as reflected in *a priori* approval by the Ethics Committee of Tongji Medical College of Huazhong University of Science and Technology. Written informed consent was obtained from each participant. Patient information was anonymized and de-identified prior to analysis.

### Data source

We applied stratified cluster sampling. Counties and county-level cities in China’s central (e.g., Jingshan, Songzi, and Dangyang in Hubei Province) and western (e.g., Danjiangkou and Meixian in Shaanxi Province) regions were designated as sample areas.

The sample size was calculated using the inappropriate admission rate in township hospitals. From existing research, the estimated inappropriate admission rate *P* is 16%, and the expected error *d* = 0.1 *P* = 1.6%, and *α* = 0.05. So,

$$ \mathrm{N} = {\left({Z}_{\alpha }/d\right)}^2 \times P\left(1\hbox{-} P\right) = {\left(1.96/1.6\%\right)}^2 \times 16\% \times \left(1\hbox{-} 16\%\right) = 2016.84 \approx 2018 $$

An additional 10% was added to the final sample, giving a total of 2220 medical records.

Township hospitals were ranked by size and those in the 4^th^ and 10^th^ places were chosen for the study. In each hospital, 250 non-delivery medical records from 2012 were randomly selected. A total of 2,100 records were collected (fewer than 250 records were available from Songzi and Dangyang). After screening, 2,044 records were found acceptable. All the sampling patients were covered by NRCMS, and had received reimbursement. Sociological characteristics such as gender, age, complications, admission information including hospital department, admitted severity, length of stay, disease category, and other data like the treatment capacity of the township hospital and the payment were collected to build a database using Epidata 3.2. Diseases were categorized using the International Classification of Diseases 10th revision (ICD-10). Additionally, NRCMS management personnel and doctors from the subject township hospitals were interviewed.

### Statistical analysis

From previous research, we identified possible determinants including gender, age, department, admitted severity, length of stay, disease category, complications and the payment method [15,17-19]. The treatment capacity of a medical institution is an important factor in inappropriate admission. The data we obtained indicate a hierarchical structure. The 2,044 records were aggregated by township hospital. The determinants for inappropriate admission to hospitals were examined using MLwiN 2.30, which was developed by the University of Bristol, UK, for multilevel binomial logistic regression analysis [33,34].

## Results

### AEP criteria

After three rounds of Delphi evaluation and adjustment, the AEP criteria were identified (Table 1). The criteria comprise nine requirements for services, such as surgery needs, and 20 indicators of disease severity, such as persistent fever over 38°C for 5 days. Patients were considered to be inappropriately admitted if their records did not fit any AEP criteria.

**Table 1:** **AEP criteria in township hospitalization**

**A.**	**Needed medical service**
1.	Need surgery or follow-up treatment within 24 hours: (1) local anesthesia or general anesthesia; and/or (2) instruments or other facilities that are only available for hospitalized patients (angiography, visceral biopsy)
2.	Treatment with varying dosage or drug on a regular basis under direct medical supervision
3.	Calculation of intake and output volume
4.	Operation to be conducted on the following day in the operating room, detailed pre-operative consultation or evaluation on the day of admission
5.	Main surgical incision and drainage nursing
6.	Quarantined patients
7.	Bedside electrocardiogram (ECG) monitoring or testing vital signs at least every 2 hours
8.	Stopping (at least once every 8 hours) or continuing oxygen inhalation
9.	Referral of post-operative recovery
**B.**	**Severity of illness**
1.	Continuous fever > 38.0°C for more than 5 days
2.	Acute confusion (coma or adiaphoria)
3.	Severe anomaly in electrolyte or blood and vigor, showing the following situations: (1) Na < 123 mEq/L or > 156 mEq/L; (2) K < 2.5 mEqt/L or > 6.0 mEq/L; (3) HCO_3_ < 20 mEq/L or > 36 mEq/L; and (4) arterial blood pH < 7.30 or > 7.45
4.	Loss of sight or hearing for 48 hours
5.	Loss of activity in any part of the body for 48 hours
6.	Excretion disorder or absence of intestinal peristalsis in the past 24 hours
7.	Active bleeding
8.	Needing blood transfusion because of bleeding
9.	Mental disorders caused by non-alcohol dependence
10.	Viscera removal or surgical wound dehiscence
11.	Pulse less than 50 times or greater than 140 times per minute
12.	Abnormal blood pressure: systolic blood pressure < 90 mmHg or > 200 mmHg and/or diastolic blood pressure < 60 mmHg or > 120 mmHg
13.	Ventricular fibrillation or acute myocardial ischemia shown by electrocardiogram (ECG) report or course log
14.	Acute blood disorder, severe medium-sized leukopenia, thrombocytopenia, leukocytosis, erythrocytosis, thrombocytosis or hemolysis-resulted symptoms
15.	Progressive acute neurological disorders
16.	Soft tissue injuries affecting basic self-care
17.	Acute myocardial infarction or cerebrovascular accident (stroke)
18.	Spinal cord lesions
19.	Lung infection above 40% or leafy lesions according to X-ray examination
20.	Hyperemesis or acute pain at acute attack by chronic diseases

The Cronbach coefficients for the three rounds of expert consultation were 0.676, 0.942, and 0.729. The applicability of the new AEP criteria was investigated by 32 experts, 28 of whom (87.5%) claimed the criteria could be used for more than 80% of the township hospital inpatients. Both the AEP criteria and clinical experts’ implicit diagnostic criteria were applied to judging 400 medical records (see Table 2). The judgments were consistent, and the kappa coefficient of the result was 0.686 (*u* = 10.34, *P* < 0.01).

**Table 2:** **Results of appropriateness of admission using AEP and EDC**

		**Admission assessed as appropriate using AEP**		**Total**
		**Yes**	**No**	
Admission assessed as appropriate using EDC	Yes	249	15	264
	No	39	97	136
Total		288	112	400

### Basic condition of NRCMS in counties

Table 3 shows how the patients from the five counties benefited from the reimbursement policies of NRCMS. These counties shared the same financing policies in 2012, in which an individual who pays 60 RMB receives a government subsidy of 240 RMB. Reimbursement takes two forms, namely, outpatient and inpatient reimbursement and the different payment schemes lead to a sizeable difference in hospitalization benefits.

**Table 3:** **NRCMS reimbursement policies in the survey areas in 2012**

**County**	**Songzi**	**Dangyang**	**Jingshan**	**Danjiangkou**	**Meixian**
**Payment**	Global budget;	Global budget;	Global budget;	Global budget;	Global budget;
	Fee for service;	Payment for single disease;	Fee for service;	Fee for service;	Payment per day;
	Limited payment for single disease	Payment per day	Payment for single disease;	Payment for single disease	Clinical pathway
			Payment by performance		
**Hospitalization reimbursement**	Deductible of 100 RMB and reimbursement ratio of 90% for township hospitals	Deductible of 100 RMB and a reimbursement ratio of 100% for township hospitals	Deductible of 100 RMB and a reimbursement ratio of 90% for township hospitals	Deductible of 100 RMB and a reimbursement ratio of 65% for township hospitals	Deductible of 150 RMB and a reimbursement ratio of 90% for township hospitals
	Deductible of 300 RMB and a reimbursement ratio of 80% for county hospitals	Deductible of 500 RMB and a reimbursement ratio of 70% for county hospitals	Deductible of 400 RMB and a reimbursement ratio of 75% for county hospitals	Deductible of 200 RMB and a reimbursement ratio of 75% for county hospitals	Deductible of 500 RMB and a reimbursement ratio of 75% for county hospitals
**Outpatient service reimbursement**	A reimbursement ratio of 40% for township health clinics and village clinics; payment limited to 10 RMB per day, and 300 RMB per year	A reimbursement ratio of 40% for township health clinics and village clinics; payment limited to 100 RMB per year	A reimbursement ratio of 25% for township health clinics and village clinics; payment limited to 6 RMB per day, and 200 RMB per year	A reimbursement ratio of 40% for township health clinics and village clinics; payment limited to 10 RMB per day, and 300 RMB per year	A reimbursement ratio of 65% for township health clinics and village clinics; no minimum is required, payment limited to 50 RMB per year per household x number of participants

### Characteristics of patients inappropriately admitted to township hospitals

Table 4 shows the characteristics and inpatient admissions data for the subject patients. A total of 26.5% of the records indicated inappropriate admissions. The incidence rate varied in different regions (*P* < 0.001), which ranged from 10% to 40%. More than half of the cases, 57.5%, were women, but appropriate admissions were only 51.7% (*P* = 0.021). The average age of the 2,044 hospitalized patients was 54.7 years, whereas the mean age of inappropriate admissions was 57.8 years (*P* < 0.001). Elderly patients (52.1%) were more likely to be inappropriately admitted (*P* < 0 .001). Patients in internal medicine departments were more likely to have been inappropriately admitted than other departments (*P* < 0.001). Inappropriate admission also occurred mostly in non-urgent patients (*P* < 0.001). Respiratory, circulatory, and skeletal muscle diseases were more likely in those inappropriately admitted (*P* < 0.001). No statistical difference between the two groups was found in terms of length of stay (LOS) and whether the patients had complications.

**Table 4:** **Distributions of characteristics of appropriateness of admission (n = 2044)**

**Variable**	**All**	**Appropriateness of admission**		***P***
		**Yes**	**No**	
		**Number (%)**	**Number (%)**	
All	2044	1503 (73.5)	541 (26.5)	
County				<0.001
Songzi	320 (15.7)	283 (88.4)	37 (11.6)	
Dangyang	323 (15.8)	194 (60.1)	129 (39.9)	
Jingshan	488 (23.9)	354 (72.5)	134 (27.5)	
Danjiangkou	476 (23.3)	280 (58.8)	196 (41.2)	
Meixian	437 (21.4)	392 (89.7)	45 (10.3)	
Gender				0.021
Male	956 (46.8)	726 (48.3)	230 (42.5)	
Female	1,088 (53.2)	777 (51.7)	311 (57.5)	
Age, years				<0.001
Less than 20	144 (7.0)	121 (8.1)	23 (4.3)	
20–39	201 (9.8)	160 (10.6)	41 (7.6)	
40–59	762 (37.3)	567 (37.7)	195 (36.0)	
More than 59	937 (45.8)	655 (43.6)	282 (52.1)	
Mean (SD)	54.7 (19.7)	53.6 (20.3)	57.8 (17.5)	<0.001
Treating department				< 0.001
Internal medicine	1,254 (61.4)	869 (57.8)	385 (71.2)	
Surgery	589 (28.8)	491 (32.7)	98 (18.1)	
Pediatrics	32 (1.6)	26 (1.7)	6 (1.1)	
Other	169 (8.3)	117 (7.8)	52 (9.6)	
Admitted severity				< 0.001
General	847 (41.4)	530 (35.3)	317 (58.6)	
Urgent	580 (28.4)	356 (23.7)	224 (41.4)	
Serious	617 (30.2)	617 (41.1)	0 (0.0)	
Disease category				< 0.001
Respiratory disease	492 (24.1)	360 (24.0)	132 (24.4)	
Urinary disease	455 (22.3)	386 (25.7)	69 (12.8)	
Circulatory disease	461 (22.6)	288 (19.2)	173 (32.0)	
Digestive disease	112 (5.5)	79 (5.3)	33 (6.1)	
Injury and poison	173 (8.5)	155 (10.3)	18 (3.3)	
Symptoms	122 (6.0)	99 (6.6)	23 (4.3)	
Bones and muscles	188 (9.2)	102 (6.8)	86 (15.9)	
Other	41 (2.0)	34 (2.3)	7 (1.3)	
Complications				0.952
No	913 (44.7)	671 (44.6)	242 (44.8)	
Yes	1130 (55.3)	832 (55.4)	298 (55.2)	
Length of stay				0.661
Less than 8 days	1077 (52.7)	797 (53.0)	280 (51.8)	
8–14 days	746 (36.5)	549 (36.5)	197 (36.4)	
More than 14 days	221 (10.8)	157 (10.4)	64 (11.8)	
Mean (SD)	8. 5 (5.3)	8.4 (5.2)	8.7 (5.5)	0.262

### Determinants of inappropriate admissions to township hospitals

The two-level logistic regression used was by MLwiN 2.30, the medical records were level 1, and the township hospitals level 2. The level 2 variance of the zero model was statistically significant (χ^2^ = 4.917, *P* = 0.027), with aggregation of information at the township level.

The specific results of the introduction of the explanatory variables to fit two variance components models are shown in Table 5. The major determinants of inappropriate admissions to township hospitals were age, hospital department, severity of illness on admission, and disease category. If other factors remain constant, inappropriate admission was higher among groups aged 20 and above. The accidental estimation of the group aged 20 and above was about 2.6, which is considerably higher than younger groups. No difference existed between pediatrics and internal medicine, and inappropriate admissions for surgical and other departments were far below those for internal medicine. More severe conditions had lower rates of inappropriate admission. Meanwhile, digestive, symptomatic, and respiratory diseases had the same rate of inappropriate admission. The rates for circulatory and skeletal muscle diseases were higher than respiratory diseases (*P* < 0.001), whereas the rates for urinary tract diseases, trauma, and poisoning were much lower (*P* < 0.001).

**Table 5:** **Multilevel logistic regression model analysis of inappropriate admission**

	**Parameter estimate**	**Standard error**	***χ*** ^***2***^	***P***	**Adjusted OR**
**Fixed part:**					
Constant	1.010	0.457	4.880	0.027	—
Age (baseline: less than 20)					
20–39	0.939	0.368	6.503	0.011	2.557
40–59	1.017	0.314	10.479	< 0.01	2.765
More than 59	0.985	0.307	10.330	< 0.01	2.678
Department (baseline: Internal medicine)					
Surgery	-1.140	0.222	26.443	< 0.01	0.320
Pediatrics	-0.109	0.564	0.037	0.847	0.897
Other	-0.750	0.259	8.376	< 0.01	0.472
Admitted severity	-1.757	0.136	165.88	< 0.01	0.173
Disease (baseline: Respiratory disease)					
Urinary disease	-0.518	0.226	5.245	0.022	0.596
Circulatory disease	0.402	0.172	5.464	0.019	1.495
Digestive disease	0.037	0.275	0.018	0.893	1.038
Injury and poison	-0.814	0.336	5.870	0.015	0.443
Symptoms	-0.297	0.293	1.026	0.311	0.743
Bones and muscles	1.301	0.271	22.97	< 0.01	3.673
Other	-0.395	0.483	0.671	0.413	0.674
**Random part:**					
Hospital variance	0.850	0.388	4.796	0.028	—
Patient scale parameter	1	0.00	—	—	—

Note: Admitted severity is included in the analysis by order of ranked data.

## Discussion

### AEP criteria

Based on the existing management capacity and supervision scope of NRCMS, AEP is the most effective regulatory tool. The AEP developed in this study is based on the service capacity and need for hospitalization in rural China. The standard applies to common diseases, and mainly covers surgery, gynecology, and pediatrics. A few specialist diseases are exceptions. A kappa coefficient of 0.686 for AEP and clinical experts’ implicit diagnostic criteria indicates high consistency. The former could reflect the results of the latter at 0.686, which is higher than other evaluated tools in previous studies [35]. From the studies published in the past 20 years, no approach could guarantee total reliability in evaluating inappropriate admission [1]. The present AEP criteria, which were developed based on the physical condition and medical needs of the general public, could be implemented in large rural areas in China.

Otherwise, it is worth noting that the standard of whether hospitalization was appropriate is based on disease development and treatment demands, but social factors were not taken into account. These might include older people living alone, the need for multiple outpatient visits, or distance from medical facilities and difficulties traveling. Although such hospitalizations are clinically unreasonable, their rationality is worth discussing from the point of view of humanization and social care.

### Reasons for aggregation of inappropriate admissions at township hospital level

The two-level logistic regression shows an evident hierarchy among the inpatient data (township hospitals–patients). Patients hospitalized at the township level are clustered. The township hospitals of different counties differ in NRCMS reimbursement, payment policies, service concept, and medical capability, which affect the incidence of inappropriate admission. Because of limited information, the results which these factors affect inpatient service reflected from a qualitative point of view.

First, the design of outpatient and inpatient NRCMS reimbursement influences the rate of inappropriate admission. International experience indicates that decreasing the out-of-pocket (OOP) share of inpatients in hospital fees induces excess hospitalization demand and increases costs [29]. If the difference between inpatient and outpatient reimbursement is not large, patients prefer inpatient treatment. For example, those paying through a family account or via individual reimbursement could afford only a few visits to the clinic, because they would have to fund any more themselves. The reimbursement for outpatients is lower and there is a limit per day, whereas the corresponding inpatient reimbursement is higher, as much as 70%. As a result, patients exaggerate their outpatient needs to enjoy a more economical inpatient service. Dangyang County uses a fund-pooling system for outpatient service, with reimbursement of up to 40% and a limit of 100 RMB per capita. The incidence of inappropriate admission reaches 39.9% because the 100 RMB only pays for a few outpatient visits, compared with inpatient reimbursement, through which all expenses exceeding 100 RMB will be reimbursed. Thus, for patients with chronic diseases, who need more frequent clinic visits, the tendency for inappropriate admission increases, up to 39.9%. Jingshan reimburses 6 RMB per outpatient, with the balance to be paid by the patient. Those suffering from chronic diseases or requiring multiple visits tend to choose hospitalization after paying 100 RMB, because 90% of inpatient costs can be reimbursed. Meixian County has no reimbursement limitations for outpatients and has a lower reimbursement for inpatient services than other counties, so residents have to pay more to be admitted. It therefore has a lower hospitalization rate than other counties, and the inappropriate admission rate is only 10.3%.

The payment modes of NRCMS determine the way in which township hospitals operate, affecting the incidence of inappropriate admission. For example, payment for single disease leads to inappropriate admissions. Township hospitals sometimes need to accept patients with minor illnesses as inpatients, to lower the average cost of hospitalization, and to get more reimbursement. Counties that pay different rates for different conditions, such as Dangyang, Jingshan and Danjiangkou, have higher rates of inappropriate admissions. Some common conditions, some regular diseases such as respiratory and circulatory problems, have higher inappropriate admission rates than others in payment for single disease.

Geographical environment correlates with inappropriate hospitalization. Meixian and Songzi County are both plains, and their rate is about 10%. Dangyang and Jingshan are hilly, and Danjiangkou is mountainous; they have higher rates, suggesting that convenience of travel to receive treatment also influences hospitalization.

The management of township hospitals also influences the appropriateness of hospitalization. Meixian County is a pilot project of the World Bank Loan/British Government Grants China Rural Health Development Project. Its township hospitals have undergone more comprehensive and standard reforms and have strong administration, translating to fewer inappropriate admissions. Danjiangkou uses a system of exclusions to control inappropriate admission, which defines 14 kinds of medical problems, 13 kinds of surgical problems, and nine other diseases that do not require hospitalization. These include where Hb > 6 g/L in iron deficiency anemia, and a liver cyst of less than 2 cm. The remaining inpatient services are reimbursed. This policy has, to some extent, caused inappropriate admissions for other diseases to rise, and it now has a higher inappropriate admission rate than any other county.

### Individual characteristics of patients who were hospitalized inappropriately

Table 4 shows that neither a patient’s experience of medical complications nor length of stay have any significant effect on inappropriate admissions, which are affected by age, treating department, admitted severity, and disease category, as shown in Table 5. The largest proportion of inappropriate admissions (52.1%) occurred among older people, which is consistent with other research [36], and children had the lowest incidence. Inappropriate admission among older people may be for two reasons. First, a high demand for health care and an inadequate outpatient account could eventually lead to hospitalization. Second, traveling can be difficult for older people, and since most of them no longer need to go to work, hospitalization is more convenient.

Medical wards tend to have distinctly more inappropriate admissions (30.7%) than surgical wards and pediatrics. Surgical cases require hospitalization, so it is easy to distinguish whether hospitalization is needed, while medical wards have less control over hospitalization standards. The lower rate of inappropriate admissions in pediatric departments (18.75%) conflicts with previous studies, where the global inappropriate admission rate among children reached 20% to 40% [31]. The disparity may be associated with parental choice. Parents may be more inclined to choose county hospitals for children, and the small number of cases, just 32, may not be representative. Inappropriate admission generally occurs among patients with minor diseases. Severely afflicted patients more often require hospitalization, and the rate is therefore more rational.

Respiratory, circulatory, digestive, and endocrine conditions have a higher incidence of inappropriate admission, while urinary and other conditions have a lower incidence [37], perhaps because of the differing treatment capabilities and control levels of outpatient departments. Urinary diseases are poorly controlled in clinics, so admissions are more appropriate. The conditions with more inappropriate admissions are those where it is not easy to differentiate whether outpatient or inpatient care is necessary. Children and older people with low immunity and resistance to disease are more vulnerable to respiratory diseases, such as influenza, bronchial pneumonia in children, and chronic bronchitis in older people. These diseases have higher occurrences and longer durations. Their symptoms usually last 5–7 days, and while medication and rest are critical to recovery, many patients find no improvement in village clinics or outpatient treatments after brief periods and request hospitalization. These diseases do not meet the admission standards, either on severity or intensity, so such admissions are considered inappropriate.

## Conclusions

Inappropriate admission to township hospitals in Chinese rural areas is high, according to the AEP. The reimbursement design and methods of NRCMS often promote inappropriate admission. Payment without checking for appropriateness, fee for service, and little difference between inpatient and outpatient reimbursement were also causes. Individual requirements, coupled with the interests of medical institutions, seem to cause inappropriate admissions. The Chinese government could control rural area admissions in two ways. First, this study suggests that improving the out-of-pocket payments would reduce inappropriate admissions [38]. In particular, it would help to differentiate NRCMS inpatient and outpatient reimbursement and reduce the economic considerations that drive patients to choose hospitalization, to make them select their treatment more rationally. Second, it would help to strengthen the admission supervision system in medical institutions. This would include clear identification standards for admission, emphasis on severity, and more focus on respiratory diseases.

## Abbreviations

NRCMS: New Rural Cooperative Medical System
AEP: Appropriateness evaluation protocol
OOP: Out-of-pocket
LOS: Length of stay
